# Insufficiency of Food in Certain Case

**Published:** 1883-10

**Authors:** 


### Insufficiency of Food in Certain Cases.

In the *Med. Press and Circular*, August 8th, is an address by Dr. Graily Hewitt, which contains the following passage:

"The period of life during which quantitative deficiencies in the dietary are most common is the two or three immediately following the arrival of puberty. The girl is at school probably; her appetite is bad, or the food is not palatable, or is deficient in important particulars, or as I have found in some cases, she eats little in order to keep thin; the strength fails, the appetite diminishes, and a habit of taking little is formed—particularly little animal food is taken. Three or four years of the most critical stages of life are thus passed—a time at which the body should be growing fast, and to maintain this growth in adequate vigor large supplies of nutritious material are required, instead of which the supply is far below the normal standard. The result is a general impairment of vigor and of vital action. On the generative organs the effect is, as I have observed in numbers of cases, most decidedly injurious; and the effect in most instances of this kind—that the tissues of the uterus lose their normal, firm, healthy consistence. The further result is that the pelvic organs as a whole, but particularly the uterus, undergo a subsidence in the pelvis; the uterus becomes liable to change of shape, and other alterations more or less marked in different cases. Slight exertions, slight accidents, or even moderate exercise, are under these circumstances liable to act most prejudicially on the softened and weakened contents of the pelvis. This is a faithful description of what I have observed to occur in very many cases. I refrain from pursuing the further history of patients so affected as not falling within the scope of these remarks."

A WRITER in *Phila. Medical Times* declares that alcoholism is unknown in Brazil, and that the cause is coffee. Cafes in which the delicious infusions of the bean are dispensed abound there, as saloons for malt and spiritous beverages abound here. A leading medical authority of Rio de Janeiro declares that the number of drunkards in a country is in inverse ratio to the amount of coffee consumed.—*Medical Times.*

